# Scaling up malaria elimination management and leadership: a pilot in three provinces in Zimbabwe, 2016–2018

**DOI:** 10.1186/s12936-020-03255-z

**Published:** 2020-05-20

**Authors:** Amanda Marr Chung, Peter Case, Jonathan Gosling, Roland Gosling, Munashe Madinga, Rudo Chikodzore, Macdonald Hove, Greyling Viljoen, Precious Chitapi, Matsiliso Gumbi, Peliwe Mnguni, Joseph Murungu, Busisani Dube, Patience Dhliwayo, Joseph Mberikunashe

**Affiliations:** 1 Malaria Elimination Initiative, Global Health Group, Institute of Global Health Sciences, University of California, San Francisco, USA; 2 Bristol Business School, University of West of England, Bristol, UK; 3 College of Business, Law & Governance, James Cook University, Townsville, Australia; 4 Business School, University of Exeter, Exeter, UK; 5 Clinton Health Access Initiative, Harare, Zimbabwe; 6 Ministry of Health and Child Care, Harare, Zimbabwe; 7 Independent Consultant, Pretoria, South Africa; 8 Independent Consultant, Harare, Zimbabwe; 9 University of South Africa, Pretoria, South Africa; 10 HEALTHQUAL, University of California, San Francisco, USA

**Keywords:** Programme management, Challenges, Leadership, Malaria elimination, Capacity building, Zimbabwe, Service delivery, Operations

## Abstract

**Background:**

Focus for improved malaria programme performance is often placed on the technical challenges, while operational issues are neglected. Many of the operational challenges that inhibit malaria programme effectiveness can be addressed by improving communication and coordination, increasing accountability, maintaining motivation, providing adequate training and supervision, and removing bureaucratic silos.

**Methods:**

A programme of work was piloted in Zimbabwe with one malaria eliminating province, Matabeleland South in 2016–2017, and scaled up to include two other provinces, Matabeleland North and Midlands, in 2017–2018. The intervention included participatory, organization development and quality improvement methods.

**Results:**

Workshop participants in Matabeleland South reported an improvement in data management. In Matabeleland North, motivation among nurses improved as they gained confidence in case management from training, and overall staff morale improved. There was also an improvement in data quality and data sharing. In Midlands, the poorly performing district was motivated to improve, and both participating districts became more goal-oriented. They also became more focused on monitoring their data regularly. Participants from all provinces reported having gained skills in listening, communicating, facilitating discussions, and making presentations. Participation in the intervention changed the mindset of malaria programme staff, increasing ownership and accountability, and empowering them to identify and solve problems, make decisions, and act within their sphere of influence, elevating challenges when appropriate.

**Conclusions:**

This pilot demonstrates that a participatory, organization development and quality improvement approach has broad ranging effects, including improving local delivery of interventions, tailoring strategies to target specific populations, finding efficiencies in the system that could not be found using the traditional top-down approach, and improving motivation and communication between different cadres of health workers. Scale-up of this simple model can be achieved and benefits sustained over time if the process is imbedded into the programme with the training of health staff who can serve as management improvement coaches. Methods to improve operational performance that are scalable at the district level are urgently needed: this approach is a possible tactic that can significantly contribute to the achievement of global malaria eradication goals.

## Background

Malaria elimination programme implementation requires a high level of efficiency and rigor to achieve set targets in the face of technical and operational challenges and a struggle to maintain financing and political support [1]. Despite the existence of national policies on paper, these do not translate to the actual practices on the ground [2]. Focus for improved programme performance is often placed on the technical challenges, while operational issues are neglected [3, 4]. Operational challenges include but are not limited to suboptimal coverage of surveillance and vector control tools, stock-outs of commodities, fuel and vehicle shortages, inadequate data management, lack of knowledge about how to implement policies or guidelines, and how to achieve community and private sector engagement [5, 6]. Many of the challenges that inhibit programme effectiveness can be addressed by improving communication and coordination, increasing accountability, maintaining motivation, providing adequate training and supervision, and removing bureaucratic silos [5]. In order to achieve malaria elimination and eventual eradication, the malaria community needs to tackle these problems, while also ensuring enabling factors for malaria programme success, such as political and financial commitment, human and financial resources, leadership, and the capacity of overall health systems, are commensurate to reach these goals [7].

According to *The Lancet* Commission on malaria eradication, “*effective management and implementation of malaria programmes are the most important requirements for national and regional elimination and eventual global eradication*”, but it is a neglected topic that does not get attention or funding from major donors [3]. To further compound the problem, operational challenges are site specific, and the national programme cannot understand and provide the granularity of strategy adjustment to suit each type of challenge faced in each locality. One possible way of addressing these local and specific problems would be to shift the focus for solving such issues to the district level, seeking out practical ways of changing operational practices to improve malaria healthcare delivery. This change in perspective is a paradigm change, and as a consequence, other behavioural and structural norms within the health system would also need to change. District managers could, for example, be empowered to find solutions using local personnel and finances. Such local identification, analysis, and response to operational challenges could be undertaken in a manner that is transparent to line management at all levels—clinic, district, provincial and national, so that actions are understood to be legitimate, necessary and proportional. These processes should also be made evident to local stakeholders affected by such actions—such as community health workers, village leaders, environmental health practitioners, and other clinic staff.

Operational challenges, can be tackled with effective management and team work, but few district or regional health management teams have the training or skills in these topics to carry this out [1, 8]. To address this problem, a scalable training and mentoring programme was developed based on change management principles, participatory organization development (OD) approaches, and quality improvement (QI) methods to improve malaria elimination operational delivery at the district, clinic, and village level in three provinces of Zimbabwe [9–17]. The design of this programme was led by University of West of England (UWE), with input from University of California, San Francisco (UCSF), drawing upon components used during an intensive, 1 week programme for experienced managers, the majority of whom are Executive MBA (Master of Business Administration) students [18, 19]. Strengthening the subnational level creates an important bridge between the national, facility, and community levels. This capacity building contributes to the attainment of outcomes and overall strengthening of a high-quality health system. A critical component of this approach is that the individuals or teams facing certain operational challenges bring these challenges to the training events and work on them with peers; and the proposed solutions become a shared agenda for the next phase. In this way, the process combines training, problem-solving and institutionalization of a collaborative way of managing. Importantly, line managers are also involved, as their contributions and buy-in are essential to this long-term improvement in management practices. By ensuring these elements are in place, capacity building for programme management can be scalable and sustainable.

## Methods

### Pilot sites

To achieve programme objectives, a collaboration was formed between the Zimbabwe Ministry of Health and Child Care (MoHCC), UCSF, UWE, Clinton Health Access Initiative (CHAI) Zimbabwe, and OD consultants from South Africa and Zimbabwe. The pilot was implemented in one malaria eliminating province, Matabeleland South in 2016–2017, scaling up to two other provinces, Matabeleland North and Midlands, in Zimbabwe during 2017–2018.

Early versions of the process improvement intervention were trialed in field work conducted in the Central Highlands of Vietnam during the 2014–2015 malaria season, with funding from the US Naval Health Research Center (Case P, University of West of England, personal communication). During the 2016–2017 malaria season, the project worked with Beitbridge and Gwanda districts in Matabeleland South province. Matabeleland South province had reoriented to focus on malaria elimination in 2012 for all of its seven districts. Beitbridge and Gwanda are the two districts with the highest malaria incidence within the province and the two remaining districts for which indoor residual spraying (IRS) was implemented [5]. From 2017 to 2018, work continued with the two districts in Matabeleland South and expanded to all seven districts in Matabeleland North province (Binga, Bubi, Hwange, Lupane, Nkayi, Tsholotsho, and Umguza) and two districts in Midlands province (Chirumhanzu and Kwekwe). Of the seven districts in Matabeleland North, five had reoriented towards malaria elimination as had both districts of Midlands.

### Activities

The programme objective was to change the mindset of district-level malaria teams to: (1) increase productivity, coverage, and quality of operations, (2) develop management capacity at the sub-national level, (3) empower frontline workers to take ownership, solve problems, and act on decisions, and (4) optimize limited resources, while integrating for efficiency with other programmes.

**Fig. 1:**
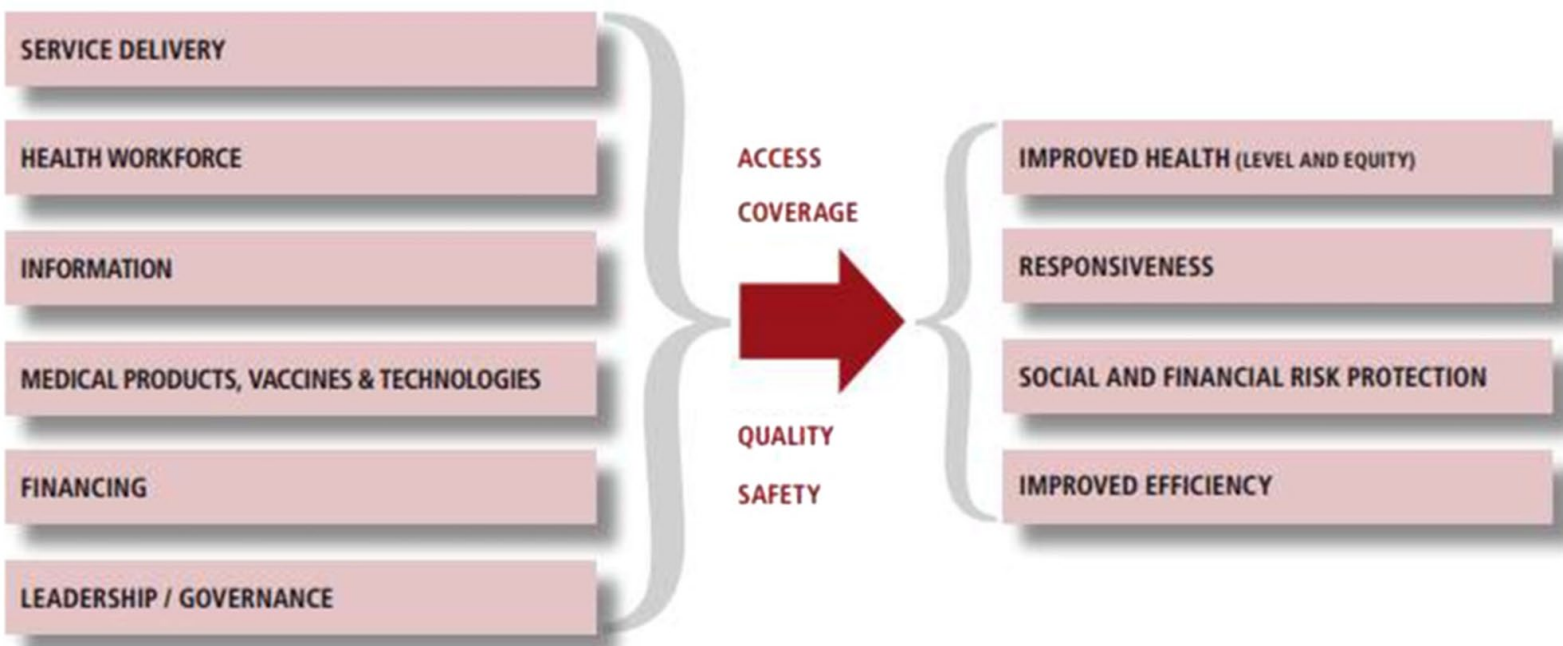
Health system building blocks from WHO’s Framework for Action [20]

The programme model seeks to strengthen all six World Health Organization (WHO) building blocks of a health system (Fig. 1), focusing directly on leadership/governance, service delivery and health information [20]. Empowerment of staff to find innovative solutions to their local problems has indirect effects on health workforce through improved motivation, optimized use of available medical products and technologies, and on financing through seeking local efficiencies in service delivery.

The intervention was based on organization development principles of building knowledge and skills within district-level malaria teams to improve effectiveness and bring about organizational change and better performance [9–13]. A participatory, action-oriented approach was also employed [16, 17]. Quality improvement methods such as root cause analysis and prioritization tools were introduced in 2017–2018 and continue in on-going activities.

**Fig. 2:**
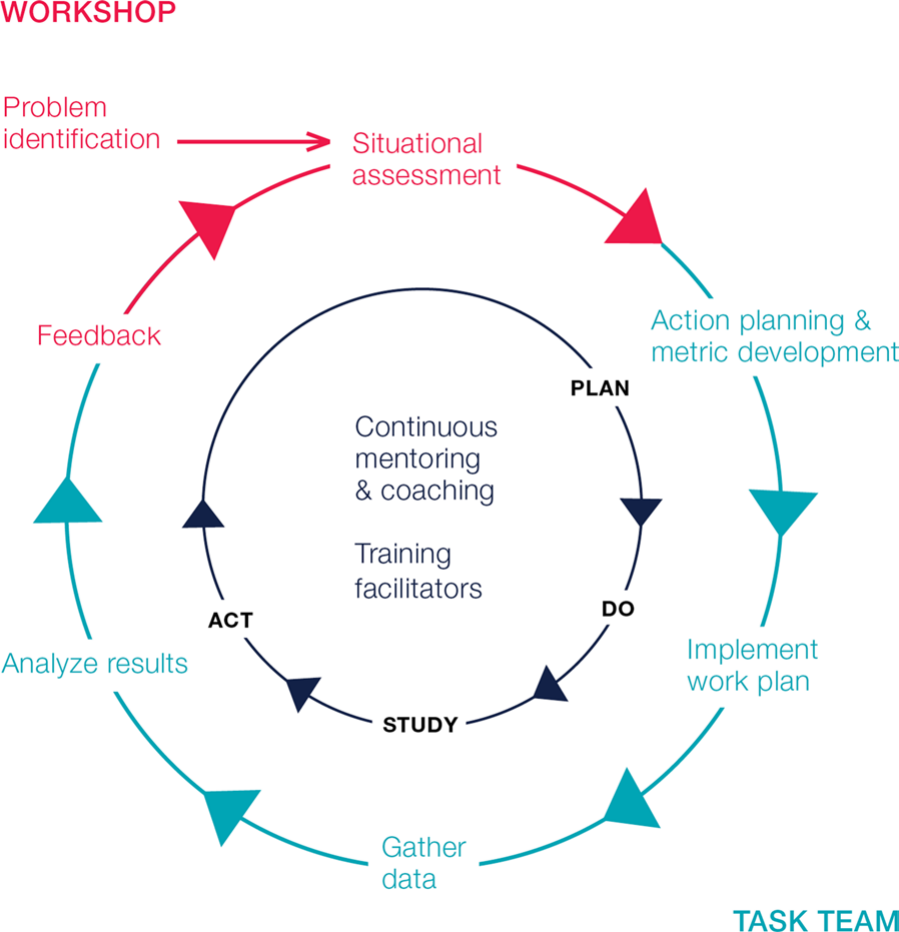
Continuous process improvement cycle

A systematic process was employed, involving continual diagnosis of challenges, action planning, implementation and evaluation to build capacity for change management. Figure 2 depicts the project cycle, which varied from 8 months to 1 year, depending on the timing of the initial workshop for each province.

During the initial workshop, an OD technique was introduced called “system in the room” to replicate the malaria programme in the meeting space. This entailed inviting participation from approximately 30–60 participants, who represented a full spectrum of disciplines and functions related to malaria control at the health facility, district, provincial, and national levels (see Table 1 for workshop participant composition). UCSF/UWE external facilitators led the workshop participants through structured exercises to identify the range of challenges they faced: from the disbursement of funding and a shortage of motorbikes to accessing flood-prone villages and community resistance to IRS. The purpose of constructing this participatory inventory of challenges was to facilitate communication and see varying perspectives on the system from different cadres and levels. These exercises aimed to focus on psychosocial elements behind the challenges each individual faced and was accomplished through asking all participants to develop graphical representations of the challenges obstructing the implementation of the malaria elimination strategy. Small groups then further analysed each individual challenge and proposed solutions to address them. Through these exercises the group identified and prioritized unresolved operational challenges that a smaller, more focused ‘Task Team’ would take forward over the course of the malaria season.

**Table 1:** Composition of workshop participants

Level	Roles
National	NMCP Deputy Director, M&E Assistant
Provincial	Administrator, MNCH Health Officer, Epidemiology and Disease Control Officer, Health Information Officer, M&E Officer, Pharmacy Manager, Health Promotion Officer, Environmental Health Officer, Accountant
District	Medical Officer, Nursing Officer, Environmental Health Officer, Health Information Officer, Pharmacy Manager, Health Promotion Officer, Lab Technician, Administrator
Health facility	Nurse, Environmental Health Technician
NGO	Technical Advisor, Associate, Analyst

The Task Team was a cross disciplinary and cross-hierarchical subset of 10–15 workshop participants comprising members from different cadres and both district and provincial levels (see Box 1 for roles and functions). Expert coaching and facilitation were provided to each Task Team to develop a work plan, which consisted of proposed solutions and associated metrics for each operational challenge in order for performance to be systematically evaluated. The focus of the selected challenges and proposed solutions was on those that could be implemented at the local level. Moreover, at the initial Task Team meeting, national level metrics were reviewed and taken into account when the Task Team reviewed priorities agreed to during the workshop to ensure that local action plans contributed to national priorities. Facilitation helped to develop greater specificity around the challenges and proposed solutions, identification of metrics, and assignment of timelines and responsibilities to individuals. The assignment of specific individuals and development of metrics ensured accountability with respect to achievement of results. The Task Team met periodically to take service delivery challenges forward, monitor progress towards targets, and incorporate new challenges and solutions as they arose. A follow-up workshop was scheduled at the end of the project cycle (some 8–10 months after the initial workshop). At this event, the wider whole-system group reconvened to evaluate progress on the challenges from the initial workshop and define new priorities to be incorporated into provincial level planning, budgeting, and reporting for the following year.

Box 1 Function and Roles of the Task Team Function of the Task Team:

- Propose actions to address challenges identified in the initial workshop.
- Identify obstacles and enablers to the implementation of the action plans.
- Monitor progress and provide feedback on the implementation of the actions.
- Attend and actively participate in meetings and workshops.

Roles of Task Team members within the malaria program at the provincial/district/health facility level included:

- Medical Officer.
- Nurse.
- Epidemiology and Disease Control Officer.
- Health Information Officer.
- Pharmacy manager.
- Accountant.
- Health Promotion Officer.
- Environmental Health Officer.
- Lab technician.

During the second year of implementation, local facilitators from within the malaria programme and CHAI who participated in the first year of the programme received leadership training with a focus on process improvement methods and organization development in order to sustain the work after external funding was no longer available. Three of the six selected for training completed a postgraduate certificate award in Professional Practice in Change Leadership (PPCL) at Bristol Business School, UWE [21]. An online handbook for facilitators was developed, and efforts were made to integrate the work into national quality improvement programmes and existing infrastructures.

### Monitoring and evaluation

Change was measured within the pilot areas by collecting baseline, midline, and endline quantitative and qualitative data. However, due to data access limitations, comparative data in non-intervention districts was not collected, nor were confounders to impact measured, such as other district, provincial or national level investments that may have driven the changes that were measured.

The measurement framework consisted of: (1) surveys for the initial and follow-up workshops to gauge overall satisfaction with the workshop and gather suggestions for improvements; (2) baseline, midline, and endline quantitative and qualitative indicators to assess whether any change had occurred for each prioritized operational challenge within the Task Team workplans; and (3) surveys to supplement the workplan data, assess whether teamwork (communication, coordination, motivation) had improved, and if participants had gained any knowledge or skills from the project. An important aspect of the evaluation and measurement process was that members of the Task Team played a significant role in deciding for themselves which indicators and metrics best matched the challenges that had been identified and prioritized by participants. The chosen metrics were often standardized to national level metrics, ensuring that data were already being collected and could be derived from official sources, though in some instances Task Teams found it necessary to generate their own measures. The key point is that there was a strong *participatory* dimension not only to the performance improvement but also to the evaluation process.

### Costs of the programme

Costs for implementing the programme in Zimbabwe were taken from the programme’s perspective to buy in the service.

## Results

### Matabeleland South province

In Matabeleland South, implementation of activities took place over 2 years, from August 2016–September 2018. In the two intervention districts (Beitbridge and Gwanda), most challenges from Year 1 were carried into Year 2 (Table 2). One challenge from the first year (liquidation of funds and implementation of activities) was resolved sufficiently for the Task Team to take on new challenges in the second year, which are outlined in Table 2.

**Table 2:** Matabeleland South province Year 1 and 2 challenges

Challenge	Solution	Year 1 change from baseline to midline	Year 2 change from midline to endline
Malaria register availability by health facilities	Audit conducted and supportive supervision visits to health facilities. Training on use of the malaria register provided to health care workers	Beitbridge: − 23% (90% to 67%). Stockouts of registers occurred during a malaria outbreak period	− 39% (67% to 28%) District needs to scale up printing during an outbreak
		Gwanda: 10% (83% to 93% or 25/30 to 28/30 health facilities)	7% (93% to 100%)
Drug stockouts	Continuous monitoring of medicine stocks by pharmacy manager	ACTs: − 16% (22% to 6%) PQ: − 4% (6% to 2%)	ACTs: 0% (6% to 6%) PQ: 0% (2% to 2%)
Case investigation rate within 3 days	Mobilize additional human resources (contract workers, students, military) for high risk areas, implement a tracker system, monitor during monthly meetings, weekly feedback to districts	10% (55% to 65% or 65/119 to 821/1265 cases investigated of cases reported)	Beitbridge: 19% (65% to 84%)
			Gwanda: 35% (65% to 100%)
Incorrect or delayed liquidation of funds and implementation of activities	Review of financial and reporting regulations, creation of planning tools, and supportive supervision visits by managers	71% (29% to 100%)	N/A

### Matabeleland North

In Matabeleland North, implementation of activities took place over the course of 10 months from November 2017 to September 2018. Although all 7 districts participated, only 5 of the districts have oriented towards malaria elimination (Table 3).

**Table 3:** New Year 2 challenges for Matabeleland South province

Challenge	Solution	Year 2 change from baseline to endline
Village health worker (VHW) recruitment and retention	Lowered qualification requirements for VHW recruitment and provided VHWs with training and support to retain them	Beitbridge: 3% (87% to 90%)
		Gwanda: 5% (83% to 88%)
Lack of inter-provincial collaboration	Use available funding from the Elimination 8’s Global Fund grant to conduct a minimum of two peer to peer province and district learning visits. The meetings will enable harmonization of surveillance and response activities between provinces and create cross-border and inter-provincial linkages	Target achieved through initiation of inter-provincial (Matabeleland South-Masvingo) and cross border meetings (Zimbabwe- Botswana) and exchange visits)
Low IRS coverage in Beitbridge district	Conducted operational research and analysed results	Planned to implement recommendations^a^ in the 2018/19 IRS campaign
Failure to conduct entomological activities	Engage the national entomologist, conduct training on larval source management (LSM) to build the capacity of the EHTs	Beitbridge achieved target of 2 entomological activities (malaria vector bionomics investigation, bioassays for testing insecticide susceptibility and training of EHTs on LSM). Gwanda had not yet initiated entomological activities
Slow uptake of Public Finance Management System (PFMS) funds	Provided on the job training and telephone consultations	Efforts to improve use of the finance system despite internet connectivity challenges resulted in improvement in fund utilization
Failure to update slide results of RDT + cases in case-based surveillance records	Developed tool to track slides received from health facilities which will allow the microscopy center to update results on case-based surveillance and relay results back to health facility	Feedback loop between lab and health facilities will be closed with the introduction and training on this tool at health facilities

^a^Recommendations from the operational research conducted to address low IRS coverage included increased community sensitization, development of IEC materials in local languages, including radio programs in local languages, recruitment of community mobilizers, early engagement and partnership of stakeholders, and better planning for IRS timing, resources, and supplies

Midlands province Implementation of activities in Midlands province in two districts, Chirumhanzu and Kwekwe, took place over 8 months from February to September 2018 (Table 4).

**Table 4:** Matabeleland North Year 1 challenges

Challenge	Solution	Year 1 change from baseline to endline
Regular review of malaria case investigation data and data management	Identify focal persons to be point of contact and follow up for surveillance related work, provide mentoring and supportive supervision visits on how to use DHIS2 tracker (malaria elimination surveillance system) to the focal persons, regular onsite data verification and data cleaning, initiation of regular surveillance meetings	8% increase in malaria slide examination rates of confirmed cases (81% to 89% or 115/142 to 90/101 slides examined out of total positive cases) 10% increase in fully investigated cases (88% to 98% or 125 cases investigated out of 142 RDT + cases to 99/101) Weekly disease surveillance reports shared with province: 1 out of 5 districts; quarterly district review meetings conducted: 2 out of 5 districts; quarterly provincial meetings conducted: 2 Improvements to data discrepancies and timeliness (no quantitative data available to support)
Implementation of new treatment guidelines	Mentoring of health workers and VHWs, refresher trainings, setting up a help line	12% increase in the administration of primaquine (63% to 75% or 90/142 to 76/101 cases administered PQ/total positive cases) Refresher trainings ongoing but all five eliminating districts have conducted post-training follow-up visits and VHWs now trained
Coordination across departments	Map stakeholders, attend district and provincial social services and local governance meetings, develop service improvement plan	Target achieved at district level for external coordination. See Box 2. Improvements to internal coordination were in progress
Lack of ownership and accountability	Conduct team building, award best performing district, provide peer support visits	Peer support visits implemented in 2 of 5 districts. Other activities were in progress
Malaria commodity stockouts	Create reporting template for tracer commodities, supportive supervision visits to improve stock management, redistributing excess commodities to other districts, supply VHWs with essential commodities	20% improvement in medicine stock status (50% to 70%)
Larval source management	Order biolarvicide, map active breeding sites, train environmental health practitioners in LSM, use standardized bio larviciding reporting form	1 of 5 districts have completed mapping. Training in 2 of 5 districts conducted
Poor quality IEC materials, unknown effectiveness of SBCC activities	Identify translators and correct malaria messages, evaluate impact of activities	Work in progress

**Table:** Box 2 District level multi-sectorial collaborations achieved through OD/QI activities

District	Organizations	Relevant Activities or Resources
Binga	Save the Children, Binga Rural Development Committee, Anglican Diocese of Matabeleland, Wild4Life, ActionAid	IRS support, food for IRS teams, fuel
Bubi	Plan International, Mary Ellen, Bubi Rural Development Committee, Isabella Mine, Streak Farm, Joe Trading, Inyathi Training Institute	Fuel, mosquito nets, food for IRS teams, allowances, IRS truck maintenance, storage facilities for LLINs before distribution
Hwange	ZAPMI, Hwange Colliery, Global Fund, World Vision, Wild4Life Health, Dept of National Parks and Wildlife	IRS support, support for mentorship visits to health facilities, protective clothing, food for IRS teams
Lupane	World Vision, Plan International, Sizimele, I-TECH, Africa Project, Lead and COSV	IRS support, entomology activities, SBCC activities, transportation, human resources, food for IRS teams
Nkayi	World Vision, Mbuma Mission Hospital, ZRP-Nkayi, HEFO	Food for IRS teams, transportation, servicing IRS vehicles, fuel
Tsholotsho	Plan International, DDF, TRDC, Global Fund, I-TECH	IRS and LLIN distribution, net storage, transportation, mentorship and training
Umguza	Plan International, DDF, URDC, I-TECH	IRS and LLIN distribution, storage storage facilities for LLINs before distribution, transportation

### Additional qualitative results

Feedback from workshop participants was collected with a qualitative instrument for all provinces with an average completion rate of 70%. Workshop participants in Matabeleland South reported an improvement in data management, with the development of a data collection tool, the initiation of data reporting from district to province on a weekly basis, and the establishment of a data focal point in each district. In Matabeleland North, motivation among nurses improved as they gained confidence in case management from training, and overall staff morale was impacted positively. There was also an improvement in data quality and the frequency with which data was shared via weekly bulletins. In Midlands, the poorly performing district was motivated to improve, and both participating districts became more goal-oriented. They also became more focused on monitoring their data regularly and learned how to develop indicators to measure the process improvement changes they were making. Participants from all provinces reported having a better appreciation of the value of communication, teamwork, planning, continuous monitoring of data, and adjustment of work plans and gained skills in listening, communicating, facilitating discussions, and making presentations (Table 5).

**Table 5:** Midlands Year 1 challenges

Challenge	Solution	Year 1 change from baseline to endline
Treatment of confirmed malaria cases with ACTs	Supportive supervision, community training	Chirumhanzu: 7% (93% to 100%) Kwekwe: 11% (89% to 100%)
Case investigation rates	Submission of weekly disease surveillance meeting reports with action taken	Chirumhanzu: 0% (100% to 100%) Kwekwe: 19% (80% to 99%)
Poor data quality	Implemented a weekly surveillance meeting and bulletin	Improvements to data quality, completeness, and timeliness with weekly review of data in 72% of facilities in Chirumhanzu and 100% of facilities in Kwekwe
Inadequate LSM	Identify and map breeding sites, train locals on scooping and environmental modification	Chirumhanzu: 78% of sites identified and mapped (1/73 sites to 58/73 sites)
Lack of knowledge about foci management	Train EHPs, identify and map foci, conduct contact screening and treatment	2 contacts identified, screened, and treated during initiation of foci investigation activities

More importantly, participation in the intervention changed the mindset of malaria programme staff, increasing ownership and accountability, and empowering them to identify and solve problems, make decisions, and act within their sphere of influence, elevating challenges when appropriate. These changes were demonstrated by the following comments:

*“We don’t think outside the box; we create the box.”*

Senior Environmental Health Officer

*“Significant improvement (was) seen on malaria elimination indicators for both Kwekwe and Chirumhanzu. The districts became more aware of their performance and actively made efforts to improve.”*

Senior provincial-level clinician

*“(In Zimbabwe), we found that participants really appreciated the opportunity to get in a room together to discuss challenges and identify their own solutions to these challenges … the act of getting people together in one place to discuss their challenges was seen as a major accomplishment in itself.”*

Southern Africa regional malaria programme officer

*“Soon enough, people get it. By giving them autonomy, peers to reflect with, and experts to advise them on their planning, they gain confidence. The conversations change. No longer am I confronted with challenges like “we can only achieve this if we have more money”. Instead the teams work on practical solutions by reviewing together and coming up with solutions that are feasible to execute, and within their control.”*

Senior provincial-level clinician

There was also a desire to continue the work beyond the funded implementation period:

*“We have to continue this project because in Matabeleland North and Midlands, we saw the difference we made when we used the principles of Organization Development for Malaria Elimination. Let’s keep up the good teamwork, let’s communicate, let’s keep the good coordination because we can implement this project and integrate it into other activities.”*

Senior provincial-level clinician

An indirect effect of the OD/QI programme was the use of learned techniques in other areas of challenge within the health system. Box 3 describes how one trainee used change leadership skills to overcome transportation challenges in her province.

Box 3 Application of change leadership within a province

- Challenge
- Prior to undergoing UWE Professional Practice in Change Leadership training, the Provincial Medical Director had attempted to overhaul the transport management system for over a year. Vehicles were often not available due to frequent breakdowns, and there was inadequate fuel for programme activities and poor checks and balances in managing the vehicles and fuel.
- Solution
- Rather than the top-down approach she had previously taken, she empowered an administrator to research other provinces’ perspectives on the advantages and disadvantages of using vehicle trackers. This designee then reported on his recommendations and was put in charge of implementing the new system.
- Results
- After implementing the new system, the province had greater control over the availability of vehicles, vehicles were returned with adequate fuel, and there was better adherence to transport policies. The Provincial Medical Director successfully applied what she had learned during her change leadership training to encourage full participation, mutual understanding, an inclusive solution, and shared responsibility in implementing the process improvement to the transport management system.

### Costs of the programme

The cost for training within-programme facilitators was separated out. All costs were calculated in United States Dollars (USD) standardized to 2019. The breakdown of costs are shown in Table 6. The total cost of the 2-year programme was $381,134, with the average cost per district in Year 2 being $26,450. Depending on what level of outside support is needed costing estimates per district for implementation range from approximately $30,000 using non-accredited local staff within Zimbabwe and as much as $50,000 using international consultants. The average cost of $26,450 is derived over the 2-year period where in the second year most facilitation and mentoring was supported by local trained team members. Costs of training and certifying 6 trainees is shown in Table 7.

**Table 6:** Costs of 2-year programme

	Year 1 (2016–2017) 1 province, 2 districts	Year 2 (2017–2018) 3 provinces, 11 districts	Total (%)
Workshop costs (hotel, per diems)	$22,176	$100,007	$122,183 (32%)
Task Team costs (hotel, per diems)	$6608	$30,183	$36,791 (10%)
Consultant costs	$32,715	$106,259	$138,974 (36%)
Travel	$14,195	$9163	$23,358 (6%)
Project management, M&E	$14,485	$45,343	$59,828 (16%)
Total	$90,179	$290,955	$381,134

**Table 7:** Costs of change leadership programme

	Year 2 (2017–2018)	(%)
Workshop costs (hotel, per diems)	$2752	8
Consultant costs	$19,725	61
Certificate costs^a^	$10,000	31
Total	$32,477	100

^a^Costs for certification from University of West of England, UK

## Discussion

In order to meet global malaria targets stated in the WHO Global Malaria Technical Strategy, malaria programmes globally must take on and overcome operational challenges at the lowest administrative levels [22]. In this pilot project, a scalable, effective and affordable method was demonstrated to improve hard outcomes, such as increased case investigation rates and prevention of medicine stockouts amongst others, as well as improving softer, but equally important, outcomes including communications and motivation. Over 2 years in three provinces in Zimbabwe the programme showed that improving the effectiveness of a malaria programme through a participatory, action-oriented OD approach can result in significant operational improvements even over one malaria season. The intervention was iterative, strengthened by incorporating QI prioritization tools into the methods in the second year of implementation.

Key to the success of the pilot was the ownership and empowerment of the district level leaders to identify and solve operational challenges within their control. Insights into the challenges in implementing a well-articulated strategy from all actors within the health system are instructive. People at all levels, in all functions, understood the operational differences between strategies aimed at malaria control and those aimed at elimination. The implementation challenges were less universally appreciated, perhaps because the solutions are usually site-specific and dependent on local knowledge and relationships. Some examples are:

1. Hard-to-reach populations are extremely varied, each requiring specific approaches to be sustained over several years, and often dependent on the quality of relationships between key individuals in those populations and in district-level health specialists (environmental health practitioners and village health workers are especially important—both occupying positions at the lowest level of the professional status hierarchy).
2. IRS refusals are a common feature in many at-risk populations—in some cases, people locked their houses, left their village, and let the guard dogs loose when they saw the spray teams approach. While a solution in one village might be to leverage family relationships with a village leader, in another it could be entirely different: working with a school teacher to reach parents through educating their children or persuading the pastor of a church to support the IRS campaign.
3. Some people—enough to be a reservoir to sustain transmission of the disease—are actively opposed to medical intervention on religious grounds or because they are involved in illegal activities and avoid contact with formal health providers. These challenges can be tackled only by local staff empowered and resourced to do so.
4. Operational implementation is often achieved only through inventive coordination across functions and levels. For example, when pre-printed malaria registers were destroyed by unseasonal rain penetrating a storeroom, make-shift versions were created by the health facility using torn-up cereal packets, then photographed and sent by WhatsApp to the peripheral team. Unfortunately, some columns of information were left off these foreshortened versions, resulting in incomplete follow-up with patients who tested positive. Village-level information to fill these gaps enabled considerable success. Similarly, in order to sustain a fleet of motor bikes for environmental health practitioners, mechanics pooled resources from several funding streams (HIV funds for inner tubes, TB funds for driver training, MNCH funds for fuel, and malaria funds for valves and brake-pads). Necessary flexibility and trust amongst provincial, district and facility managers was much enhanced by participation in the workshops and the ability to refer to the cross hierarchal Task Teams.

Some other lessons learned include the need to develop better indicators to measure change over time. For example, the Midlands Task Team was not able to demonstrate successful implementation of their solutions to address late detection and reporting of outbreaks, low use of LLINs, and late treatment seeking by the community. In Matabeleland North and Midlands, quantitative data was not gathered to show improvements in data quality and completeness. Another lesson was the importance of securing national and provincial leadership endorsement for success. In Zimbabwe, having strong support at the national level and participation by National Malaria Control Program (NMCP) officials in the follow-up workshop led to the expansion of the work to other provinces and continuation in the existing provinces. Strong provincial level support in Matabeleland South resulted in the incorporation of Task Team challenges into routine supervisory visits and the continued implementation of work plan activities, even without support from an outside facilitator. It is also essential to have national representation in follow-up workshops to hear results and unresolved challenges and important to identify alternative sources for funding to ensure continuation of activities well in advance of the end of the implementation period.

How does this method differ from other methods of team building and quality improvement? This programme is unique from the approach taken by organizations working at the community level in that it involves all levels of the system (national, provincial, district, community), facilitates communication between all levels, allows the participants to identify the operational challenges they plan to tackle, and provides coaching and a structured process for facilitating and monitoring change. This pilot programme was advantaged by the inclusion of both organization development and quality improvement experts. Learning over the 2 years of implementation led to a hybrid design that used participatory approaches to identify the major challenges to be tackled and the formation of self-selected Task Teams, with the rigor and structures of continuous quality improvement (CQI) for prioritization, indicator setting, and plan-do-study-act cycles. The study team believes that the participatory element is needed to specifically identify challenges at the local level that cannot be generated at the central level and for the motivation and efficiencies within the “system” to be found at little or no cost to the NMCP. The CQI toolbox was invaluable for providing structure once challenges were identified to prioritize, iterate, and quantify improvement. The Zimbabwe Ministry of Health and Child Care is currently expanding quality management to include malaria amongst other new areas for targeting.

### Limitations of this pilot

The impact of external influences on the programme was not assessed, for example co-investment by other agencies such as the United States Agency for International Development/President’s Malaria Initiative (USAID/PMI) or the Global Fund to Fight AIDS, TB, and Malaria that could have led to the documented improvements in performance. One of the challenges included in the 2016–2017 malaria season workplan for Matabeleland South was a vehicle shortage for environmental health technicians. Efforts were made to ensure that vehicles and motorbikes were both functional and available to meet their needs. This resulted in an increase in the availability of functional motorbikes from 22 to 51. Some of the motorbikes from an existing fleet were serviced to become functional, but in other cases, new motorbikes were purchased through Global Fund funds secured prior to the start of the project. For this reason, this challenge was not included in the workplan. Additionally, one of the collaborating partners, CHAI, was funded throughout the period in all intervention districts to improve malaria elimination. Statements from our CHAI colleagues support the causality of the pilot project: “*The project offered a platform for districts to meet, discuss challenges, and focus efforts to resolve the challenges through implementation of agreed work plans. This led to better resource mapping within districts and efficient use of existing resources to resolve challenges*.”

A second limitation is that control districts were not included as part of the pilots from which routine data could be collected as a comparison to intervention districts. Thirdly, a missing component of the work was expertise on the financing piece, in order to address challenges related to liquidation of funds and financial management. Moreover, the project costs were relatively high in the design phase. With the training of local facilitators, costs decreased in a third year of implementation. In addition, if workshops could be integrated into existing annual planning meetings, costs could be shared. Having greater control over budgets at the subnational level is a major factor in implementing solutions. Such change requires advocacy at the national level to devolve financial responsibility to the periphery. A complementary, bottom-up approach might be to pair such advocacy with capacity building to mobilize financial resources at the local level. For this reason, the solutions that the Task Teams implemented were those that they could operationalize with existing resources, and those that were not within their control were elevated to the appropriate authorities. Lastly, although IRS coverage did increase from 72 to 80% during the 2016–2017 and remained at 80% during the 2017–2018 season, the value of the programme as it impacted the delivery of key interventions such as IRS and LLINs was not assessed. During programme implementation, the districts flagged low IRS coverage as a challenge and implemented a short survey to ascertain the reasons for households refusing IRS. The information gathered could have contributed to better IRS mobilization and implementation by the spray teams. However, it would be challenging to attribute any changes in IRS or LLIN coverage to this programme alone.

### Future work

Further work to address the limitations and leverage the benefits of the programme are needed. Firstly, the participatory nature of the work lends itself to the inclusion of local stakeholders into the “system in the room”. In two new iterations of this work in Namibia and Zimbabwe, religious and community leaders and elected representatives were included in the process and were active members on the Task Team. Another group to include in future work is the varied and unsupervised private sector. There are advantages of being more inclusive and thus expanding the “system in the room”. Secondly, the OD/QI programme supports district level programmes to change operational activities over which they have control. What if the districts or provinces need to advocate for change, for example at the national level? Experience from efforts to teach district and provincial leaders skills in advocacy, planning, resource mobilization, and policy influence in the Philippines, Sri Lanka, and Thailand suggests that adding this element to our sub-national OD/QI programme would further strengthen systems for malaria elimination. Finally, for further uptake and investment in OD/QI, what evidence of effectiveness is needed by donors and governments? Conducting a controlled trial could address this question by implementing this programme in districts in a step wedge design over a 2–4 year period in two different settings, one moderate to low burden setting and one high burden setting, where implementation challenges prevent these localities from reaping the benefits of proven cost-effective interventions.

Given the urgency of overcoming the challenges outlined in *The Lancet* Commission on Malaria Eradication [3], and “bending the curve,” rolling out and scaling-up this approach should be done quickly. Several malaria programmes are interested in using this approach to address their operational challenges. However, due to donor reluctance to invest in programme management capacity building, funding to take this forward has not materialized. Ideally this could be included and then funded in country or regional Global Fund proposals or through bilateral funding with a share of the funding increasingly coming from the affected countries over time. Ultimately, the aim of this programme is to build the capacity of national or regional organizations, creating a global cadre of trained facilitators, to apply this approach to malaria and other health programmes. This could be offered through UCSF in partnership with UWE and other academic institutions. The training materials have already been developed, and UWE can offer postgraduate certification for facilitators.

## Conclusion

Implementation challenges are the biggest unaddressed problem facing malaria programmes today. Notable recent publications state the urgent need to build leadership and management capacity at the lower levels of health systems to overcome this problem [3, 23, 24]. This pilot demonstrates that a participatory, organization development and quality improvement approach has broad ranging effects, including: improving local delivery of interventions, tailoring strategies to target specific populations, finding efficiencies in the system that could not be found using the traditional top-down approach, and improving motivation and communication between different cadres of health workers. Scale-up of this simple model can be achieved and benefits sustained over time if the process is imbedded into the programme with the training of health staff (malaria and other) who can serve as management improvement coaches. Methods to improve operational performance that are scalable at the district level are urgently needed: a participatory, organization development and quality improvement approach is a possible tactic that can significantly contribute to the achievement of global malaria eradication goals.

## Data Availability

The datasets during and/or analysed during the current study available from the corresponding author on reasonable request.

## Abbreviations

CHAI: Clinton Health Access Initiative
CQI: Continuous quality improvement
HIV: Human Immunodeficiency Virus
IRS: Indoor residual spraying
LLIN: Long-lasting insecticide-treated bed nets
MBA: Master of Business Administration
MNCH: Maternal newborn and child health
MoHCC: Ministry of Health and Child Care
NMCP: National Malaria Control Programme
OD: Organization development
PMI: United States President’s Malaria Initiative
PPCL: Professional Practice in Change Leadership
QI: Quality improvement
TB: Tuberculosis
USAID: United States Agency for International Development
UCSF: University of California, San Francisco
USD: United States Dollar
UWE: University of West of England
WHO: World Health Organization

## References

[1] Gosling J, Case P, Tulloch J, Chandramohan D, Wegbreit J, Newby G (2015). Effective program management: a cornerstone of malaria elimination. Am J Trop Med Hyg.

[2] Muhindo Mavoko H, Ilombe G, da Inocêncio Luz R, Kutekemeni A, van Geertruyden JP, Lutumba P (2015). Malaria policies versus practices, a reality check from Kinshasa, the capital of the Democratic Republic of Congo. BMC Public Health..

[3] Feachem RG, Chen I, Akbari O, Bertozzi-Villa A, Bhatt S, Binka F (2019). Malaria eradication within a generation: ambitious, achievable, and necessary. Lancet..

[4] Moonen B, Cohen JM, Snow RW, Slutsker L, Drakeley C, Smith D (2010). Operational strategies to achieve and maintain malaria elimination. Lancet.

[5] Muchena G, Dube B, Chikodzore R, Pasipamire J, Murugasampillay S, Mberikunashe J (2018). A review of progress towards sub-national malaria elimination in Matabeleland South province, Zimbabwe (2011–2015): a qualitative study. Malar J..

[6] Smith Gueye C, Newby G, Tulloch J, Slutsker L, Tanner M, Gosling R (2016). The central role of national programme management for the achievement of malaria elimination: a cross case-study analysis of nine malaria programmes. Malar J..

[7] Roll Back Malaria Partnership. Action and investment to defeat malaria 2016–2030: for a malaria-free world. 2015. https://www.mmv.org/sites/default/files/uploads/docs/publications/RBM_AIM_Report.pdf. Accessed 19 Nov 2019

[8] Wirth DF, Casamitjana N, Tanner M, Reich MR (2018). Global action for training in malaria elimination. Malar J..

[9] Easterby-Smith M, Araujo L, Burgoyne J (1999). Organizational learning and the learning organization: developments in theory and practice.

[10] Senge P (2006). The fifth discipline: the art and practice of the learning organization.

[11] Cummings T, Worley C (1993). Organization development and change.

[12] Raelin J (2003). Creating leaderful organizations: how to bring out leadership in everyone.

[13] Krznaric R (2007). How change happens: interdisciplinary perspectives for human development.

[14] NYDOH AIDS Institute. HIVQUAL Group learning guide: interactive quality improvement exercises for HIV health care providers. 2006. https://healthqual.ucsf.edu/group-learning-guide. Accessed 19 Nov 2019.

[15] NYDOH AIDS Institute. HIVQUAL workbook: guide for quality improvement in HIV care. 1999. http://nationalqualitycenter.org/files/hivqual-workbook/. Accessed 19 Nov 2019.

[16] Koch T, Kralik D (2006). Participatory action research in healthcare.

[17] Reason P, Bradbury H (2001). Handbook of action research: participative inquiry and practice.

[18] Managing around the world: roundtables for experienced managers. https://www.embaroundtables.com/. Accessed 14 Feb 2020.

[19] Gosling J, Case P (2013). Give me the answer: the paradox of dependency in Management Learning. e-Org People..

[20] WHO. Everybody’s business: strengthening health systems to improve health outcomes: WHO’s framework for action. 2007; Geneva: World Health Organization. https://www.who.int/healthsystems/strategy/everybodys_business.pdf. Accessed 19 Nov 2019.

[21] Bristol UWE—Module specifications—Professional practice in change leadership. https://info.uwe.ac.uk/Modules/displayentry.asp?code=UMODDR-60-M&rp=listEntry.asp. Accessed 22 Nov 2019.

[22] WHO. Global Technical Strategy for Malaria 2016–2030. Geneva, World Health Organization, 2015. https://www.who.int/malaria/publications/atoz/9789241564991/en/. Accessed 12 July 2019.

[23] Rabinovich RN, Drakeley C, Abdoulaye A, Djimde B, Fenton Hall SI, Hay JH (2017). MalERA: an updated research agenda for malaria elimination and eradication. PLoS Med..

[24] Cohen JM (2019). Remarkable solutions to impossible problems: lessons for malaria from the eradication of smallpox. Malar J..

